# Migración de catéter de derivación ventrículo-peritoneal distal a arteria pulmonar: una complicación poco común

**DOI:** 10.23938/ASSN.1046

**Published:** 2023-08-24

**Authors:** Pelayo Hevia-Rodríguez, Mikel Armendariz-Guezala, José Undabeitia-Huertas

**Affiliations:** Departamento de Neurocirugía Hospital Universitario Donostia San Sebastián España

**Keywords:** Hidrocefalia, Derivación ventrículo-peritoneal, efectos adversos, Fallo protésico, Migración de cuerpo extraño, Arteria pulmonar, Hydrocephalus, Ventriculoperitoneal shunt, adverse effects, Prosthesis Failure, Foreign-Body Migration, Pulmonary artery

## Abstract

La derivación ventrículo-peritoneal es un procedimiento quirúrgico habitual para eliminar el exceso de líquido cefalorraquídeo (hidrocefalia), asociado a distintas complicaciones.

Se presenta el caso de un varón de 60 años con hidrocefalia postraumática al que se le implantó una derivación ventrículo-peritoneal. Tras la mejoría clínica inicial, trece meses después desarrolló empeoramiento de la marcha y problemas cognitivos. Las radiografías y tomografía computarizada de tórax mostraron que el catéter distal de la derivación había migrado a la arteria pulmonar. Se extrajo quirúrgicamente el catéter mediante reapertura de la incisión retroauricular previa y tracción manual, sin incidencias. Se implantó un nuevo catéter peritoneal con mejoría clínica inmediata. Dos años después, el paciente permanece asintomático.

Este caso ilustra una complicación infrecuente de un procedimiento neuroquirúrgico habitual que puede ser detectada por diferentes profesionales sanitarios; revisamos sus diferentes formas de presentación y estrategias de manejo multidisciplinar a partir de diecinueve casos similares publicados.

## Abstract (en)

Ventriculoperitoneal shunt placement is a common treatment for hydrocephalus, although not devoid of complications.

We report a case of a 60-year-old male who underwent ventriculoperitoneal shunt implantation for the treatment of post-traumatic hydrocephalus. Thirteen months post- surgery, after an initial clinical improvement, the patient manifested gait and cognitive disorders. Chest X-rays and computed tomography revealed that the distal shunt catheter had migrated into the pulmonary artery. The catheter was removed by reopening the previous retroauricular incision followed by manual traction, without incidents. A new peritoneal catheter was implanted with immediate clinical improvement and no further complications two years after the second surgery.

We communicate a rare complication of a standard neurosurgical procedure that can be detected by different healthcare professionals, and review its various forms of presentation and multidisciplinary management strategies from 19 similar clinical cases found in the literature.

## INTRODUCCIÓN

La derivación de líquido ventricular es un procedimiento quirúrgico habitual para el tratamiento del acúmulo de líquido cefalorraquídeo (LCR) en los ventrículos cerebrales (hidrocefalia) de diversas etiologías^1^. Los sistemas de derivación normalmente se componen de un catéter proximal insertado en el sistema ventricular cerebral, un sistema intermedio regulador de presión o *válvula*, y un catéter distal que drena el LCR que exceda dicha presión a una cavidad intracorpórea.

El tipo de derivación más utilizada es la ventrículo-peritoneal (DVP), desde el ventrículo lateral hasta la cavidad peritoneal, si bien existen otras opciones como las derivaciones ventrículo-atrial, ventrículo-cisternal, ventrículo-pleural, ventrículo-biliar, ventrículo-ureteral/vesical o lumbo-peritoneal^1^. Durante la implantación de DVP se realiza el paso del catéter distal con un tunelizador subcutáneo desde el abdomen hasta el punto de acceso ventricular, en dirección craneal. En caso de que no se pueda realizar la tunelización directa, es frecuente realizar un paso intermedio mediante una incisión cervical lateral; su inserción en peritoneo se realiza mediante una mini-laparotomía.

Se estima que la cuarta parte de los pacientes sometidos a una DVP desarrollarán alguna complicación a lo largo de su vida, la mayoría durante el primer año tras la implantación^2^, que puede ocurrir en cualquier punto a lo largo de su curso desde el ventrículo cerebral hasta la cavidad peritoneal. La complicación más frecuente es la obstrucción con malfunción valvular secundaria. Una de las complicaciones más infrecuentes es la migración del catéter fuera del peritoneo, generalmente a localizaciones infradiagmáticas^1^^,^^3^^-^^5^: cavidad torácica, vejiga, hígado, intestino, vagina, o escroto.

Presentamos el caso de un paciente que trece meses tras implantarle una DVP sufrió una extraña migración del catéter a la arteria pulmonar, provocando una disfunción valvular. El catéter fue retirado mediante tracción manual desde su inserción proximal, sin otras complicaciones.

## CASO CLÍNICO

Varón de 60 años con diagnóstico de hidrocefalia postraumática que requirió de la implantación de una DVP. La tomografía computarizada (TC) craneal y las radiografías de trayecto valvular postoperatorias confirmaron la correcta colocación del catéter proximal en el ventrículo y una trayectoria adecuada del catéter distal hasta peritoneo, experimentando una mejoría clínica postquirúrgica inmediata.

**Figura 1:**
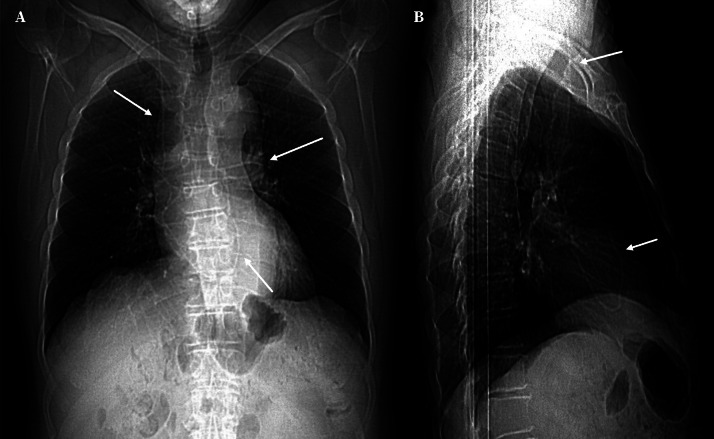
Radiografías de tórax anteroposterior (A) y lateral (B) donde se observa el recorrido del catéter hasta introducirse en cavidades cardíacas derechas y arteria pulmonar (flechas blancas).

**Figura 2:**
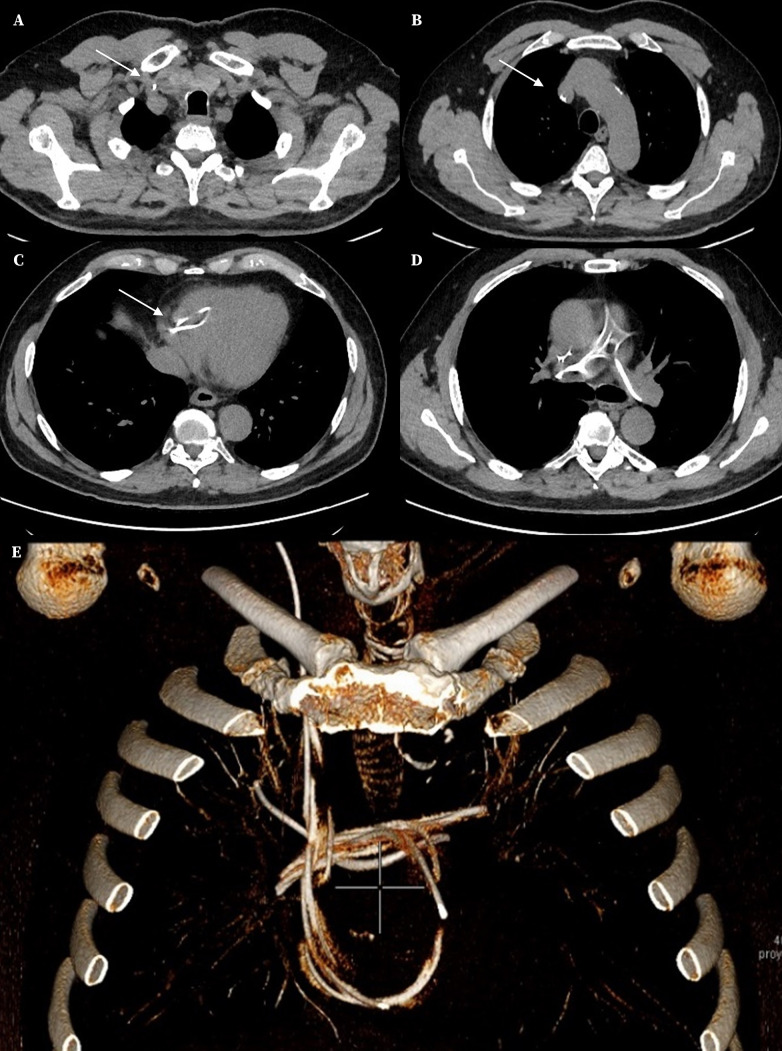
Tomografía computarizada torácica con reconstrucciones en 3D. Se observa el trayecto del catéter (flechas blancas): entrada por venta yugular interna (**A**), continuación por vena cava superior (**B**), atrio derecho - ventrículo derecho (**C**), tronco pulmonar y, tras varias vueltas en ambas arterias pulmonares (**D**), el extremo distal se aloja en la arteria segmentaria anterior (**E**).

Trece meses después, el paciente sufrió un nuevo deterioro clínico con afectación de la marcha, de la memoria, y de las funciones ejecutivas. Se realizaron radiografías del trayecto valvular. En la radiografía de tórax se localizó el catéter distal a nivel de cavidades derechas y arteria pulmonar (Fig. 1), observación confirmada mediante una TC torácica (Fig. 2).

Se intervino quirúrgicamente, procediendo a la reapertura de la incisión retroauricular previa para extraer el catéter mediante tracción manual en sentido proximal, sin incidencias. Se implantó un nuevo catéter a nivel peritoneal con mejoría clínica inmediata. El paciente recibió enoxaparina a dosis profilácticas durante el ingreso hospitalario de cinco días, sin desarrollo de tromboembolismo. Dos años después, el paciente se mantiene estable clínicamente y no ha vuelto a presentar complicaciones.

## DISCUSIÓN

La migración del catéter a la arteria pulmonar es una complicación infrecuente tras la implantación de un procedimiento quirúrgico tan frecuente como la DVP.

Se realizó una búsqueda en la base de datos PubMed, mediante los términos “*migration*” (migración), “*shunt*” (derivación) y “*pulmonar artery*” (arteria pulmonar). Se incluyeron los casos de migración de catéter de derivación hacia el corazón y/o la vascularización pulmonar, escritos en inglés o español, y se excluyeron las derivaciones ventrículo-auriculares. Se identificaron diecinueve casos clínicos publicados entre 1994 y 2023 que cumplían los criterios de selección, cuyas características y manejo se analizaron^3^^,^^4^^,^^6^^-^^10^^,^^12^^-^^23^ (Tabla 1).

**Tabla 1:** Características de los casos de migración de catéter de derivación ventrículo-peritoneal a arteria pulmonar identificados en PubMed

Estudio	Paciente	Catéter	Complicaciones relacionadas
- Autoría	- Edad (años), sexo	- Punto de entrada	
- País	- Causa de hidrocefalia	- Método de retirada	
- Año	- Tiempo hasta diagnóstico	- Reposición; momento	
	- Síntomas		
- Morell y col^7^	- 12, hombre	- VY derecha	Extrasístoles ventriculares y bradicardia durante la tracción
- EEUU	- Mielomeningocele	- Endovascular	
- 1994	- 4 años	- Sí: nuevo, DVP; inmediato	
	- Cefalea, vómitos		
- Kubo y col^12^	- 48, hombre	- VYE derecha	-
- Japón	- Post HSA	- Incisión cervical	
- 2002	- 1 mes	- Sí: mismo, DVP; inmediato	
	- Dolor cervical		
- Rodríguez-Sánchez y col^13^	- 38, hombre	- VY	Arritmia
- España	- Obstructiva	- Endovascular	
- 2003	- 1 año	- Sí: nuevo, DVP; inmediato	
	- Alteración marcha		
- Fewel y col^14^	- 16, hombre	- VYE derecha	-
- EEUU	- Post-traumática	- Incisión retroauricular	
- 2004	- 1 mes	- Sí: nuevo, DVP; inmediato	
	- Crisis		
- Chong y col^15^	- 68, mujer	- VYI derecha	Fallo DVA
- Korea	- Post-HSA	- Endovascular	
- 2008	- 2 semanas	- Sí: mismo, DVA; inmediato (retirada percutánea)	
	- Dolor abdominal		
- Hermann y col^3^	- 51, mujer	- VYE derecha	-
- Alemania	- HNT	- Endovascular	
- 2009	- 5 meses	- Sí: nuevo, DVP; inmediato	
	- Incidental		
- Ryugo y col^16^	- 50, hombre	- VYI derecha	-
- Japón	- Post-HSA	- Incisión cervical	
- 2009	- 2 años	- Sí: mismo, DVP; tras 2 días	
	- Infección herida abdominal		
- Ruggiero y col^17^	- 14, mujer	- VYI derecha	-
- Italia	- n/e	- Incisión cervical	
- 2010	- 1 mes	- Sí: mismo, DVA; inmediato	
	- Dolor abdominal		
- Nguyen y col^18^	- 28, hombre	- VYI derecha	-
- EEUU	- Idiopática	- Endovascular	
- 2010	- 8 meses	- Sí: nuevo, DVP; n/e	
	- Dolor cervical		
- Nordbeck y col^19^	- 6, hombre	- VY izquierda	Deterioro valvular derecho, arritmias
- Alemania	- Rotura de quiste aracnoideo	- Toracotomía y venotomía	
- 2010	- n/e	- Sí: nuevo, DVP; n/e	
	- Fiebre, disnea, soplo cardíaco		
- Zairi y col^20^	- 63, hombre	- VYI derecha	-
- Francia	- Obstructiva	- Incisión retroauricular	
- 2012	- 1 semana	- Sí: nuevo, DVP; n/e	
	- Incidental		
- Aboukais y col^21^	- 30, hombre	- VYI derecha	Apertura de vena cervical por bloqueo
- Francia	- Post-meningitis	- Incisión cervical	
- 2015	- n/e	- Sí: nuevo, DVP; inmediato	
	- Incidental		
- Lyon y col^6^	- 71, hombre	- n/e	-
- EEUU	- HNT	- Incisión cervical	
- 2016	- 3 semanas	- Sí: nuevo, cervical contralateral; tras 2 meses	
	- Tríada Hakim-Adams		
- Dossani y col^8^	- 30, hombre	- n/e	Rotura de catéter distal, retirada endovascular
- EEUU	- Congénita	- Retroauricular y endovascular	
- 2017	- 1 mes	- Sí: nuevo, DVP; tras 1 día	
	- Cefalea, vértigos		
- Ralston y col^9^	- 7, hombre	- n/e	-
- EEUU	- Obstructiva	- Toracotomía y venotomía	
- 2017	- 10 años	- No	
	- Insuficiencia cardíaca derecha		
- Hajdarpasic y col^10^	- 56, hombre	- VYE derecha	Endocarditis
- Bosnia-Herzegovina	- Obstructiva	- Incisión cervical	sepsis
- 2019	- 3 años	- No	
	- Fiebre persistente		
- Li y col^22^	- 19, hombre	- VY derecha	-
- China	- Obstructiva	- Toracotomía y venotomía	
- 2019	- 2 meses	- Sí: nuevo, DVP; inmediato	
	- Alteración de la marcha		
- Adib y col^23^	- 38, hombre	- n/e	-
- Alemania	- Obstructiva	- Endovascular	
- 2020	- 7 meses	- Sí: nuevo, DVP; inmediato	
	- Resfriado, dolor torácico		
- González-Pombo y col^4^	- 68, hombre	- VYE derecha	-
- España	- Post-traumática	- Sin retirada	
- 2022	- 8 años		
		- no (hallazgo incidental)	

DVA: derivación ventrículo-atrial; DVP: derivación ventrículo-peritoneal; HNT: hidrocefalia normotensiva del adulto; HSA: hemorragia subaracanoidea; VY: vena yugular; VYE: vena yugular externa; VYI: vena yugular interna; n/e: no especificado.

En la bibliografía revisada predominaban los pacientes de sexo masculino (78,9%) y con media de edad de 37,5 años (rango de 6 a 71). Se ha observado una mayor frecuencia en adultos (79%) que en pacientes pediátricos (21%).

Se han propuesto dos posibles mecanismos de migración intravascular principales^6^^,^^7^. Durante la cirugía se puede producir una perforación iatrogénica directa de las venas yugulares internas o externas durante el proceso de tunelización, daño que puede pasar completamente desapercibido o manifestarse como un hematoma cervical postoperatorio. El segundo mecanismo, que debido a los movimientos repetitivos de flexión-extensión del cuello, el catéter erosiona la pared venosa. La vena yugular interna es particularmente vulnerable a este fenómeno debido a su gran tamaño y pared relativamente delgada^8^. Una vez que el catéter está dentro del sistema venoso, la presión inspiratoria junto con el flujo sanguíneo ortógrado conduce a la migración del catéter de derivación distal al lado derecho del corazón y, posteriormente, a las arterias pulmonares^6^^,^^8^.

El tiempo transcurrido desde la cirugía hasta la detección de la migración varía desde una semana hasta diez años^9^ (Tabla 1). En la mitad de los casos, la migración ocurrió dentro de los primeros dos meses, lo que sugiere una perforación venosa directa, frente a la otra mitad de mayor tiempo de evolución, que sugiere una erosión venosa por la movilidad cervical repetida. Nuestra hipótesis es que la migración del catéter en el paciente del caso presentado corresponde al segundo mecanismo; ya que no desarrolló ningún hematoma cervical tras la intervención, y la detección de la migración se produjo trece meses después.

La forma de presentación clínica es muy variada. El síntoma más habitual es la disfunción valvular (31%), ya sea en forma de hidrocefalia aguda (cefalea, vómitos, crisis) o crónica (alteración de la marcha, disfunción cognitiva, afectación de esfínteres). En el 21% de los casos la migración del catéter se detectó de forma incidental. En casos más graves puede complicarse por la formación de émbolos pulmonares o sepsis^10^. Nuestro paciente sufrió una regresión a la sintomatología de hidrocefalia previa a la colocación de la derivación, aunque mantenía un buen estado general, sin signos clínicos de sepsis ni hallazgos de embolismo pulmonar en las pruebas de imagen.

La estrategia de manejo debe ser individualizada y multidisciplinar. En todos los casos revisados se optó por la retirada del dispositivo excepto en el publicado por González-Pombo y col^4^, que fue un hallazgo incidental en un paciente asintomático. El catéter fue recolocado de nuevo en el 84% de los casos, y retirado de forma definitiva en el 10%. La estrategia más sencilla, y por la que optamos en nuestro caso, es la retirada del catéter distal mediante tracción manual. Debemos revisar con detenimiento las pruebas de imagen, buscando algún posible nudo o bloqueo que pueda causar problemas de rotura u obstrucción. Recomendamos realizar la retirada desde la propia incisión retroauricular, para poder comprobar la conexión más proximal al sistema de derivación, con la ayuda o no de una incisión cervical. En caso de correcto funcionamiento, y en ausencia de nudos o roturas del catéter y de infección subyacente, se puede reintroducir el mismo catéter u optar por uno nuevo. Si bien la estrategia más común es repetir la inserción peritoneal, también existe la opción de realizar una reconversión a derivación ventrículo-atrial o de una retirada definitiva del sistema si sospechamos que el paciente puede no requerir ya del sistema. En cualquier caso, se debe intentar realizar una trayectoria nueva para minimizar el riesgo de nueva lesión venosa. A la hora de la retirada es importante prever las posibles complicaciones que pueden surgir durante y después el procedimiento. Durante su manipulación, el catéter se puede romper, siendo necesario un rescate endovascular percutáneo. También se puede bloquear en cualquier punto del trayecto por la presencia de un nudo, requiriendo de una apertura cervical y/o toracotomía para su extracción^9^.

Durante y después de la cirugía se pueden producir arritmias, lesión cardíaca o vascular y formación de tromboembolismos. Por estos motivos, recomendamos el apoyo en quirófano de los equipos de Radiología Intervencionista y de Cirugía Cardiovascular, el uso de ecocardiografía transtorácica durante el procedimiento, y una vigilancia post-anestésica con monitorización cardíaca continua de al menos 24 horas. No existen unas pautas definidas sobre el manejo de anticoagulación profiláctica en estos pacientes, por lo que la elección debe ser individualizada teniendo en cuenta el resto factores de riesgo protrombóticos. En nuestro caso, dada la ausencia de arritmias cardíacas durante la monitorización intra y postoperatoria, así como la ausencia de otros factores de protrombóticos, decidimos administrar únicamente enoxaparina 40 mg/24h profiláctica durante el ingreso, sin observarse la formación de trombos.

Para tratar de prevenir esta complicación, se debe analizar la anatomía de las venas superficiales del cuello, y evitar realizar una tunelización del catéter demasiado medial y profunda. La mayoría de los autores no notaron ningún sangrado anormal durante la implantación de la derivación que podría alertar sobre una perforación yugular^4^. Por protocolo en nuestro centro realizamos radiografías de trayecto valvular a todos los pacientes tras una derivación ventricular. Se debe tener en cuenta que, incluso si el paciente presenta un sangrado cervical intraoperatorio, es posible que en una radiografía simple no se vean anomalías. Para aquellos casos en los que existe un alto sospecha de perforación yugular, se debe considerar realizar una TC cérvico-torácica. Existen autores que en caso de sangrado cervical recomiendan realizar un seguimiento mediante radiografías de tórax repetidas, sin especificar durante cuánto tiempo^10^. Sin embargo, la realización de radiografías seriadas durante mucho tiempo en ausencia de síntomas clínicos puede provocar una acumulación de radiación considerable^11^, teniendo en cuenta que se trata de una complicación poco común.

La migración del catéter distal de derivación ventrículo-peritoneal a la arteria pulmonar es una complicación rara. Puede ser causa de malfunción valvular, entre otra sintomatología. La presentación de este caso muestra una manera sencilla y efectiva de solucionar el problema. A partir de la revisión de la literatura realizada, aportamos una serie de consideraciones a tener en cuenta a la hora de la retirada del catéter con el fin de evitar posibles complicaciones durante y después del procedimiento. Es posible que una detección y retirada rápida disminuyan el riesgo de desarrollar arritmias o tromboembolismo.

## References

[1] Greenberg MS (2022). Manual de Neurocirugía.

[2] Merkler AE, Ch’ang J, Parker WE, Murthy SB, Kamel H (2017). The rate of complications after ventriculoperitoneal shunt surgery. World Neurosurg.

[3] Hermann EJ, Zimmermann M, Marquardt G (2009). Ventriculoperitoneal shunt migration into the pulmonary artery. Acta Neurochir.

[4] González-Pombo M, Torri JA, Olivares Blanco M (2022). Catéter de derivación ventrículo-peritoneal migrado a la arteria pulmonar: revisión de la literatura a propósito de un caso. Neurocirugía.

[5] Taub E, Lavyne MH (1994). Thoracic complications of ventriculoperitoneal shunts: case report and review of the literature. Neurosurgery.

[6] Lyon K, Ban VS, Bedros N, Aoun SG, El Ahmadieh TY, White J (2016). Migration of a ventriculoperitoneal shunt into the pulmonary vasculature: case report, review of the literature, and surgical pearls. World Neurosurg.

[7] Morell RC, Bell WO, Hertz GE, D’Souza V (1994). Migration of a ventriculoperitoneal shunt into the pulmonary artery. J Neurosurg Anesthesiol.

[8] Dossani RH, Maiti TK, Patra DP, Nanda A, Cuellar H (2017). Endovascular retrieval of migrated distal end of ventriculoperitoneal shunt from bilateral pulmonary arteries: a technical note. Ann Vasc Surg.

[9] Ralston A, Johnson A, Ziemer G, Frim DM (2017). Transcardiac migration of ventriculoperitoneal shunt requiring open cardiac surgery: case report and review of the literature. Childs Nerv Syst.

[10] Hajdarpašić E, Džurlić A, Mahmutbegović N, Zahirović S, Ahmetspahić A, Arnautović K (2019). Sepsis caused by bacterial colonization of migrated distal ventriculoperitoneal shunt catheter into the pulmonary artery: a first case report and literature review. World Neurosurg.

[11] Wall BF, Haylock R, Jansen JTM, Hillier MC, Hart D, Shrimpton PC (2011). Radiation risks from medical X-ray examinations as a function of the age and sex of the patient.

[12] Kubo S, Takimoto H, Takakura S, Iwaisako K, Yamanaka K, Hosoi K (2002). Peritoneal shunt migration into the pulmonary artery - case report. Neurol Med Chir (Tokyo).

[13] Rodríguez-Sánchez JA, Cabezudo-Artero JM, Porras Estrada LF (2003). Unusual migration of the distal catheter of a ventriculoperitoneal shunt into the heart: case report. Neurosurgery.

[14] Fewel ME, Garton HJL (2004). Migration of distal ventriculoperitoneal shunt catheter into the heart. Case report and review of the literature. J Neurosurg.

[15] Chong JY, Kim JM, Cho DC, Kim CH (2008). Upward migration of distal ventriculoperitoneal shunt catheter into the heart : case report. J Korean Neurosurg Soc.

[16] Ryugo M, Imagawa H, Nagashima M, Shikata F, Hashimoto N, Kawachi K (2009). Migration of distal ventriculoperitoneal shunt catheter into the pulmonary artery. Ann Vasc Dis.

[17] Ruggiero C, Spennato P, De Paulis D, Aliberti F, Cinalli G (2010). Intracardiac migration of the distal catheter of ventriculoperitoneal shunt: a case report. Childs Nerv Syst.

[18] Nguyen HS, Turner M, Butty SD, Cohen-Gadol AA (2010). Migration of a distal shunt catheter into the heart and pulmonary artery: report of a case and review of the literature. Childs Nerv Syst.

[19] Nordbeck P, Beer M, Wirbelauer J, Ritter O, Weidemann F, Ertl G (2010). Intracardial dislocation of a cranio-peritoneal shunt in a 6-year-old boy. Clin Res Cardiol.

[20] Zairi F, du Moulinet d’Hardemaere V, Assaker R (2012). Early cardiac migration of distal shunt catheter. Br J Neurosurg.

[21] Aboukais R, Zairi F, Marinho P, Lejeune JP (2015). Management of cardiac migration of a distal shunt catheter: the radiological pitfalls. Neurochirurgie.

[22] Li W, Li Y, Sun Y, Chen L (2019). Migration of a distal ventriculoperitoneal shunt catheter into the pulmonary vasculature: a report of an unusual case and a review of the literature. J Craniofac Surg.

[23] Adib SD, Lescan M, Renovanz M, Schuhmann MU, Trakolis L, Bongers M (2020). Intracardial catheter migration of a ventriculoperitoneal shunt: pathophysiology and interdisciplinary management. World Neurosurg.

